# The Application of Social Characteristic and L1 Optimization in the Error Correction for Network Coding in Wireless Sensor Networks

**DOI:** 10.3390/s18020450

**Published:** 2018-02-03

**Authors:** Guangzhi Zhang, Shaobin Cai, Naixue Xiong

**Affiliations:** 1 Computer Science Department, Harbin Engineering University, Harbin 150001, China; caishaobin@hrbeu.edu.cn; 2 Department of Information Engineering, Suihua University, Suihua 152000, China; 3 Computer Science Department, Huaqiao University, Xiamen 361021, China; 4 Department of Computer Science, Georgia State University, Atlanta, GA 30302, USA; dnxiong@ieee.org

**Keywords:** network coding, error propagation, error correction, L1 optimization, social network

## Abstract

One of the remarkable challenges about Wireless Sensor Networks (WSN) is how to transfer the collected data efficiently due to energy limitation of sensor nodes. Network coding will increase network throughput of WSN dramatically due to the broadcast nature of WSN. However, the network coding usually propagates a single original error over the whole network. Due to the special property of error propagation in network coding, most of error correction methods cannot correct more than *C*/2 corrupted errors where *C* is the max flow min cut of the network. To maximize the effectiveness of network coding applied in WSN, a new error-correcting mechanism to confront the propagated error is urgently needed. Based on the social network characteristic inherent in WSN and L1 optimization, we propose a novel scheme which successfully corrects more than *C*/2 corrupted errors. What is more, even if the error occurs on all the links of the network, our scheme also can correct errors successfully. With introducing a secret channel and a specially designed matrix which can trap some errors, we improve John and Yi’s model so that it can correct the propagated errors in network coding which usually pollute exactly 100% of the received messages. Taking advantage of the social characteristic inherent in WSN, we propose a new distributed approach that establishes reputation-based trust among sensor nodes in order to identify the informative upstream sensor nodes. With referred theory of social networks, the informative relay nodes are selected and marked with high trust value. The two methods of L1 optimization and utilizing social characteristic coordinate with each other, and can correct the propagated error whose fraction is even exactly 100% in WSN where network coding is performed. The effectiveness of the error correction scheme is validated through simulation experiments.

## 1. Introduction

Wireless Sensor Networks (WSN) suffer many constraints such as limited battery energy, low transmission rate and poor-quality links [1]. How to provide a reliable data transmission in WSN in order to prolong the network lifetime as long as possible is an important and challenging issue. A new attractive technique named network coding goes especially well with WSN due to the broadcast nature and diversity of the links in WSN [2]. Network coding will reduce the number of transmissions and receptions in network nodes which results in the reduction of energy consumption. Network coding is a technique where relay nodes mix and combine packets using mathematical operations, which reduces the number of transmitted packets. Network coding was firstly proposed in [3]. The traditional architecture of networks is storing-and-forwarding, and it was once believed that switch and router would not bring any benefit. However, it turns out that network coding can increase the network throughput dramatically which results in a high packets delivery ratio. Even in the application, network coding also has a good performance [4]. Some works also intend to reach the lower bound of the theory bandwidth as far as possible, for example, by constructing new codes [5]. In summary, network coding has already seen tremendous advancement in both theory and application.

Although network coding can dramatically improve the performance of WSN, it suffers a serious disturbance from propagated errors due to its inherent mixture characteristic. In network coding, its very nature of combining information in the relay nodes makes the network very susceptible to transmission errors. A single error will be propagated by every node further downstream in the networks. This will thus prevent the reconstruction of the file in the sink. The poor-quality links in WSN further intensify the crisis of error propagation in network coding. There are fruitful works about network error correction coding for network coding (NEC) [6,7,8,9]. However, none of the existing works thoroughly solved the error propagation problem in network coding. Most of the pre-existing works about NEC have apparent drawbacks. Homomorphic signature scheme which is based on Cryptographic approaches has high complexity and intolerable delay [10]. NEC which is based on information theoretic approaches has low complexity, but they cannot cope with the dense propagated errors which exceed *C*/2 where *C* is the max flow min cut of the multicast network. Some works are based on hamming distance [11,12,13,14] is based on hamming distance and other works are based on rank distance [15] is based on rank distance; Neither escapes the above rule. Guangzhi’s works increase the information rate as far as possible, the effectiveness of such improvements is also limited [16,17]. Koetter and Kschischang present a seminal idea of subspace codes without having to consider the randomness of the random network, and it is a huge progress [18]. However, subspace codes still cannot correct errors which exceed *C*/2 [18]. Due to the nature of the linear block codes from which NECs are developed, *C*/2 is the upper bound of the corrected errors number. The number of corrected errors is bounded by Shannon information theory. Thus, the constraint means the number of links where an error occurs cannot exceed *C*/2. However, it is unrealistic to assume the number of original error is less than *C*/2. The number of links, where “original error” occurs, usually exceeds *C*/2. Furthermore, the number of random errors caused by channel noise, is usually very large. Under a fixed bit error rate (BER), the more links there are, the more corrupted packets there will be. The situation will be worse where no link-layer error correction is performed, such as in wireless sensor networks of which the computational power is limited. If network coding theory is to be applied into practice from the theory in laboratory, it is critical to find a new error correction mechanism to cope with the propagated error in network coding. It is time to think outside the constraints of the current error correction mechanism to find brand new thinking from other research fields.

Recently, researchers have made great progress in the field of social networks and L1 optimization and sparse learning [19,20]. Based on L1 optimization, John and Yi propose a dense error correction method which can correct nearly 100% of the corrupted observations which may seem surprising and unbelievable at first observation [21]. The powerful error correcting ability of [21] naturally reminds us to introduce it to correct the dense propagated error in network coding. There are also some works applying the theory of social network to improve the performance of WSN [22,23,24]. With the two emerging techniques, hopefully, we will arrive at a solution to the thorny problem of error propagation in network coding.

Although John and Yi’s scheme looks as though it could solve the error propagation problem, it fails in the last mile. In addition, although John and Yi’s L1 optimization model can correct dense error where nearly (not exactly) 100% of the corrupted observations can be corrected, it cannot correct the propagated errors in network coding which usually pollute exactly 100% of the received messages. If we can bring down the fraction of propagated error to a little below 100%, this problem goes away. However, it is not a simple matter of reducing the fraction of error propagation in the network. The works as [11,12,13,14] are the most important works about NEC, but none of them can reduce the fraction of propagated errors. By introducing a secret channel and a matrix which can trap some errors, we successfully decrease the fraction of propagated error to a little less than 100%. The first method is using a secret channel to transmit a small part of messages in advance which will indirectly bring down the fraction of propagated error to a little less than 100%. The secret channel method requires simple and straightforward thinking, but it is very effective. The secret channel will not cost a lot of resources [25] because the percentage of messages which need to be sent can be very low, for example, 1%. As long as the faction of ultimate errors are not equal to 100%, John and Yi’s scheme can work. The second method is to set the bottom of the coding matrix in John and Yi’s scheme as an identity matrix. The original uncompressible message is added with many zeroes in the bottom part to form a sparse message vector. The sparse message is then coded with a coding matrix which is constructed based on John and Yi’s scheme. Because we know a priori that the bottom part of the sparse message vector is all zeroes, we can trap a part of errors from the received messages. This will indirectly decrease the rate of the corrupted messages. With the two novel methods, John and Yi’s L1 optimization model successfully solves the error propagation problem in network coding.

Although John and Yi’s scheme can ultimately solve the error propagation problem after our two improvements, its fraction of successful decoding is too low while the fraction of errors is in the range of between 0.85 to 1. If the received messages are not “informative” enough, the severe trend could worsen. In the context of network coding whose decoding algorithm is solving equations other than L1 optimization, the statement that “the received messages are informative” refers to the famous “all-or-nothing” problem [26]. In a sink, if the *C* received messages are full rank, we can decode successfully based on the method of solving equations, and we call the received messages “informative” or “innovative”. The sink cannot recover any information from received data, unless it receives at least the same number of innovative packets as were originally combined together. However, in the context of network coding whose decoding algorithm is L1 optimization other than solving equations, the “all-or-nothing” problem will be partially alleviated. Even if the received packets are not full rank, there is also a potential to decode the original error with L1 optimization rather than solving the equation. The property of alleviating the “all-or-nothing” problem in our scheme is similar to that of [26], referring to the compress sensing. However, the more the rank of received packets is, the greater the opportunity for the L1 optimization to decode successfully. The objective is to have relay nodes receive more “informative” or “innovative” packets from upstream nodes. However, it is not an easy task in the random environment to receive more “informative” packets. For a relay node, the definition of “informative” in the context of L1 optimization does not merely refer to having the full rank as far as possible. Even if all the relay nodes will transmit packets with full rank to the downstream nodes, the received packets are not exactly full rank. Providing the full rank of packets from upstream nodes is a local optimum solution other than the global optimum solution. If some upstream nodes whose packets not only are full rank for themselves but also make multi-hops away downstream nodes have full rank of packets, we say such upstream nodes are more “informative”. We can use one acknowledgement message from multi-hops away downstream nodes to identify which upstream nodes are more “informative”. The point is that we cannot use acknowledgement messages all the time due to their high resource consuming nature. The reputation-based trust model of the social network research field will help us to find the upstream nodes that have more opportunities to be informative for any time other than one time. If a relay node in network coding, identifies which upstream nodes that can bring more “informative” packets in advance, the received packets by the sink will have more opportunities to be full rank. The more acknowledgement characters (ACKs) received by a relay node, the more the trust value is. After the trust computation stage, we can select the optimal relay nodes to perform network coding, and other relay nodes will not perform network coding to save energy. Selecting relay nodes with high trust value will help the received packets of the sink node to be full rank, therefore L1 optimization will have more opportunities to decode successfully.

The main contribution of this paper is:

- Many 0 values are added to the original information to make the signal sparse; re-organize the transmitted signal from vector to matrix. The two methods allow L1 optimization method to be applied in network coding; therefore, L1 optimization technique can be introduced from the research field of image recognition to the research field of communication.
- The coding matrix in the original model by John and Yi is replaced by a specially designed matrix in which the bottom is identity matrix. The specially designed matrix can trap some errors and make these trapped errors known by the sink. The method will indirectly put down the fraction of propagated errors a little because we can know some errors a prior through the trapped errors.
- We use a secret channel to transmit a small amount messages in advance which will indirectly bring down the fraction of propagated errors slightly below 100%. Based on this method, John and Yi’s model can correct propagate errors in network coding.
- We propose a new distributed approach that establishes reputation-based trust among sensor nodes in order to identify the informative upstream sensor nodes. This will help L1 optimization have more opportunities to decode successfully and it will result in short delays and high throughputs.

The remainder of this paper is organized as follows. Section 2 presents a brief review on [21], and gives some basic definitions about network coding. In Section 3 and Section 4, we will formally give our scheme about error correction method in WSN which is based on L1 optimization and social networks respectively. Then, Section 5 performs experiments. Finally, Section 6 presents our conclusions.

## 2. Preliminaries and Related Works

### 2.1. Network Coding and Its Fundamental Concepts

**Figure 1:**
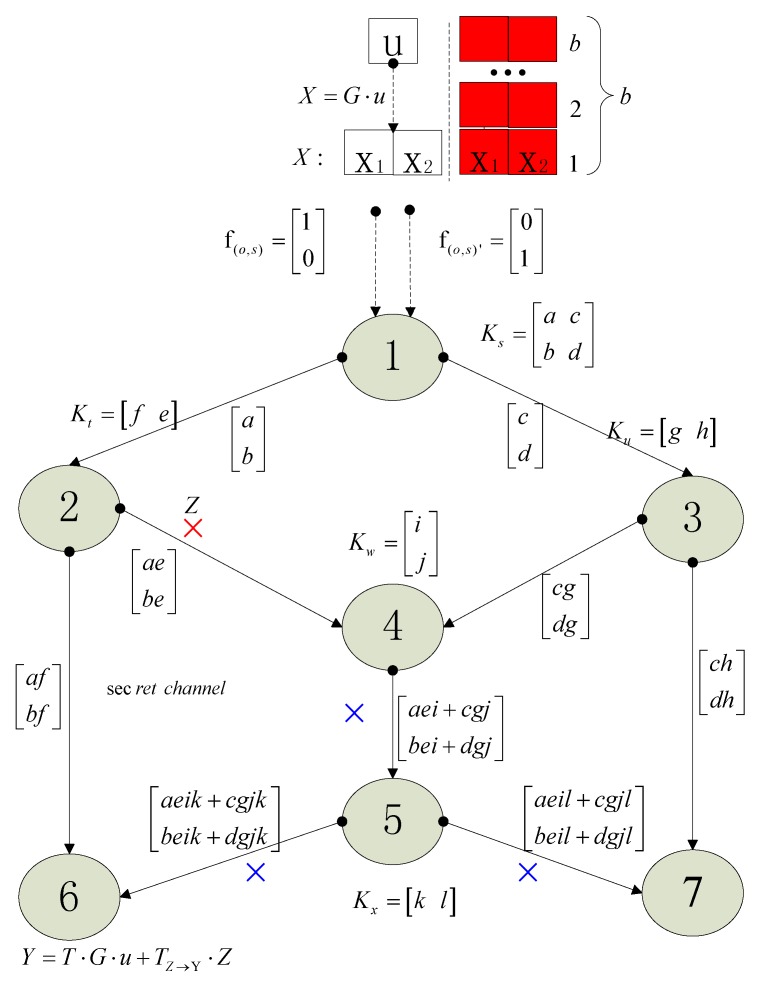
Network coding on butter-fly network. We illustrate the concept of “propagated error” with red and blue symbol “×”. The red part shows the concepts about block transmission in network coding.

Based on Figure 19.3 in [27], we give an enhanced version of this sketch map and many new elements are added. Using the new Figure 1, we will illustrate some important concepts of network coding about NEC which are referred to in this paper. These concepts are necessary for understanding the following Algorithm 1. Some related works are also referred to and a short introduction about them will be given based on Figure 1. Because of space limitation, we will not pursue a strict definition and instead give a descriptive statement. The precise definitions of concepts are illustrated in the referred background papers.

Figure 1 is the famous butter-fly picture in the research field about network coding. If the network is error-free, the source node 1 wants to multicast a message vector $X={\left[X_{1}\;X_{2}\right]}^{T}$ to both sinks 6 and 7. Some concepts are list below.

Dimension of network coding: It is equal to the max flow min cut of the multicast network. In Figure 1, the dimension denoted as w which is the size of X, therefore 2.

Coding field: The coefficients of network coding are selected from the finite field which is denoted as $F_{q}$ whose size is q. Variables a, b, …, l, which are coding coefficients, take value from $F_{q}$. All the messages including u, X, Y, Z, and all the messages in the relay nodes take value in an extension field $F_{Q}$ with size Q. G takes value in $F_{Q}$ while T and $T_{Z\to Y}$ take values in $F_{q}$.

Local coding kernel: A feasible linear network coding scheme consists of a scalar $k_{d,e}$, called the local encoding kernel, for every adjacent (d,e). The |In(v)|×|Out(v)| matrix $K_{v}={\left[k_{d,e}\right]}_{d\in In(v),e\in Out(v)}$ is called the local encoding kernel at node v. $K_{s}=\left[\begin{array}{l} a\;c\\ b\;d \end{array}\right]$, $K_{t}=\left[f\;e\right]$, $K_{u}=\left[g\;h\right]$, $K_{w}=\left[\begin{array}{l} i\\ j \end{array}\right]$, $K_{x}=\left[k\;l\right]$ are all local coding kernels.

Global coding kernel: A feasible linear network coding scheme also consists of a column vector $f_{e}$ for every channel e such that: (1) The vector $f_{e}$ for imaginary channels e∈In(s) form the standard basis of the vector space $F_{q}{}^{w}$; (2) $f_{e}=\sum_{d\in In(v)}k_{d,e}f_{d}$ for e∈Out(v). Global coding kernel and local coding field are two different mathematical descriptions, i.e., two sides of the same coin. They can be deduced from each other. $X\cdot f_{e}=X\sum_{d\in In(v)}k_{d,e}f_{d}=\sum_{d\in In(v)}k_{d,e}(X\cdot f_{d})$.

**Algorithm 1.** LOECNC AlgorithmStep 1Set involved parameters in L1 optimization according Equations (2) and (3).Step 2In real field $F_{Q}=R$, add n−k zeros to κ behind of it, to form $x_{0}\in R^{n\times 1}$. Get $y\in R^{(m+n)\times 1}$ based on $y=\left[\begin{array}{l} A\\ I_{n} \end{array}\right]\times x_{0}$. The coding matrix is as the matrix in Equation (6).Step 3In a finite field $F_{q}$, perform encoding procedure of network coding scheme in every relay node.Step 4Divide the vector y into two parts: $y=\left[\begin{array}{l} y_{A}\\ y_{B} \end{array}\right]$ where $y_{A}\in R^{(\lambda \cdot C)\times 1}$ and $y_{B}\in R^{(m+n-\lambda \cdot C)\times 1}$. To adapt to the transmission through networks, reorganize the vector $y\in R^{(m+n)\times 1}$ to the matrix $y\prime \in R^{C\times \frac{m+n}{C}}$ where $y\prime =[y\prime_{1},\ldots y\prime_{j},\ldots y\prime_{\frac{m}{C}}](j=1,2,\ldots ,\frac{m+n}{C})$ and $y\prime_{j}\in R^{C\times 1}\;(j=1,2,\ldots ,\frac{m+n}{C})$. Divide the matrix y′ into two parts: $y\prime =[y\prime_{A}\;;\;y\prime_{B}]$ where $y\prime_{A}=[y\prime_{1},\ldots ,y\prime_{\lambda }]$ and $y\prime_{B}=[y\prime_{\lambda +1},\ldots ,y\prime_{\frac{m+n}{C}}]$. Send the matrix $y\prime_{A}\in R^{C\times \lambda }$ through a secret channel. Send the matrix $y\prime_{B}\in R^{C\times (\frac{m+n}{C}-\lambda )}$ through the networks where the networks coding is performed in the relay nodes. Each time, $y\prime_{j}\in R^{C\times 1}\;(j=\lambda +1,\lambda +2,\ldots ,\frac{m+n}{C})$ which is a column of $y\prime_{B}$, is sent with network coding method. The matrix $y\prime_{B}$ is needed to be sent $\frac{m+n}{C}-\lambda$ times.Step 5In the sink, receive the matrix $y\prime_{A}=[y\prime_{1},\ldots ,y\prime_{\lambda }]$ through a secret channel and the matrix $Y_{B}$ through the networks where the networks coding is performed. $Y_{B}=[Y_{\lambda +1},\ldots ,Y_{\frac{m+n}{C}}]$ responds to $y\prime_{B}=[y\prime_{\lambda +1},\ldots ,y\prime_{\frac{m+n}{C}}]$. After the network coding effect and errors effect, $y\prime_{B}$ will become $Y_{B}$. Based on Equation (11), we know the received message responding $y\prime_{j}$ is $Y_{j}=\hat{T}\cdot y\prime_{j}+T_{Z\to Y}\cdot \left(Z-L\cdot y\prime_{j}\right)\;(j=\lambda +1,\lambda +2,\ldots ,\frac{m+n}{C})$.Step 6Perform network coding decoding algorithm to $Y_{B}=[Y_{\lambda +1},\ldots ,Y_{\frac{m+n}{C}}]$. In the finite field $F_{q}$, perform decoding of the network coding scheme in every sink to get ${\hat{T}}^{-1}$. In the real field R, compute ${\left(y\prime_{j}\right)}^{d}={\hat{T}}^{-1}\cdot Y_{j}\;(j=\lambda +1,\lambda +2,\ldots ,\frac{m+n}{C})$ in random network, where ${\left(y\prime_{j}\right)}^{d}$ is the estimate of $y\prime_{j}$. After $\frac{m}{C}-\lambda$ times, we get ${\left(y\prime_{B}\right)}^{d}=[{\left(y\prime_{\lambda +1}\right)}^{d},\ldots ,{\left(y\prime_{\frac{m+n}{C}}\right)}^{d}]\;(j=\lambda +1,\lambda +2,\ldots ,\frac{m+n}{C})$ where ${\left(y\prime_{B}\right)}^{d}$ is the estimate of $y\prime_{B}=[y\prime_{\lambda +1},\ldots ,y\prime_{\frac{m+n}{C}}]$. Reorganize the matrix ${\left(y\prime_{B}\right)}^{d}\in R^{C\times (\frac{m+n}{C}-\lambda )}$ to the vector ${\left(y_{B}\right)}^{d}\in R^{(m+n-C\cdot \lambda )\times 1}$.Step 7Reorganize the matrix ${\left(y\prime_{B}\right)}^{d}\in R^{C\times (\frac{m+n}{C}-\lambda )}$ to the vector ${\left(y_{B}\right)}^{d}\in R^{(m+n-C\cdot \lambda )\times 1}$. Divide ${\left(y_{B}\right)}^{d}$ into two parts and it is ${\left(y_{B}\right)}^{d}=\left[\begin{array}{c} {\left(y_{\left(m+n-C\cdot \lambda )-(n-k\right)}\right)}^{d}\\ {\left(y_{n-k}\right)}^{d} \end{array}\right]$. Set ${\left(y_{n-k}\right)}^{d}=0^{(n-k)\times 1}$. Update ${\left(y_{B}\right)}^{d}=\left[\begin{array}{c} {\left(y_{\left(m+n-C\cdot \lambda )-(n-k\right)}\right)}^{d}\\ 0^{(n-k)\times 1} \end{array}\right]$.Step 8In the sink, receive the matrix $y\prime_{A}$ and then reorganize the matrix $y\prime_{A}\in R^{C\times \lambda }$ into the vector $y_{A}\in R^{(\lambda \cdot C)\times 1}$.Step 9Reorganize the vector $y_{A}\in R^{(\lambda \cdot C)\times 1}$ and the vector ${\left(y_{B}\right)}^{d}\in R^{(m+n-C\cdot \lambda )\times 1}$ into $y^{d}=\left[\begin{array}{c} y_{A}\\ {\left(y_{B}\right)}^{d} \end{array}\right]$. Up on $y^{d}$, in the real field R, perform L1 optimization which is based on John and Yi’s scheme to get y.Step 10Select the first k symbols of y as κ.


Decoding for network coding: In a sink, for example, sink 6, the decoding matrix is $\left[\begin{array}{l} af\;aeik+cgjk\\ bf\;beik+dgjk \end{array}\right]$. If the vectors $\left[\begin{array}{l} af\\ bf \end{array}\right]$ and $\left[\begin{array}{l} aeik+cgjk\\ beik+dgjk \end{array}\right]$ are linearly independent or the matrix $\left[\begin{array}{l} af\;aeik+cgjk\\ bf\;beik+dgjk \end{array}\right]$ are full rank, we say, the network coding is decodable.

Feasible network coding: If all the sinks can perform a successful decoding, we say, the constructed network scheme is a feasible network coding scheme. That means $\left[\begin{array}{l} af\;aeik+cgjk\\ bf\;beik+dgjk \end{array}\right]$ and $\left[\begin{array}{l} aeil+cgjlch\\ beil+dgjldh \end{array}\right]$ need to both be full rank simultaneously which is not easily satisfied and we have to select the values of a, b, …, l delicately in the field $F_{q}$.

Transfer matrix: The propagation effect of the network coding between two nodes, whose distance is multi-hop distance, will results in a matrix transformation. The generated matrix is called transfer matrix. For example, while the transfer matrix from source node 1 to sink 6 is considered, it is $T=\left[\begin{array}{l} af\;aeik+cgjk\\ bf\;beik+dgjk \end{array}\right]$ with respect to coded message vector X, and it is T⋅G with respect to the original message u. The computing method is complicated and readers are invited to the referred paper [28].

Propagated error: we illustrate the concept of “propagated error” with red and blue symbol “×”. In the link from node 2 to node 4, where an original error Z occurs, and it is considered a 1×1 error vector, then it is marked with a red “×”. Because node 4 and 5 both select randomly coding coefficients, the original error Z will be combined into the messages along the downstream links which are link 4–5, 5–6 and 5–7. The errors in the downstream links due to the original error are marked with blue “×” in Figure 1. With respect to link 2–4 and sink 6, the transfer matrix is the left part of matrix $K_{w}\times K_{x}$ which is [ik]∈1×1. Thus, the error transfer $T_{Z\to Y}$ is [ik]∈1×1. With respect to original error Z, the propagated error in sink 6 is $T_{Z\to Y}\cdot Z$. In a similar way, with respect to link 2–4 and sink 7, the transfer matrix is [jl]∈1×1.

*Block transmit*: the red part in Figure 1 shows the concepts about block transmission in network coding. Block transmission will bring down the overhead of network coding.

### 2.2. Error-Correcting Model in John and Yi’s Model

The flowing definitions mainly refer to [21]. Consider the problem of recovering a sparse signal $x_{0}\in R^{n}$ from highly corrupted observations $y\in R^{m}$:

(1) $$y=Ax_{0}+e_{0}$$

where $e_{0}\in R^{m}$ is a sparse vector of errors of arbitrary magnitude. The model for $A\in R^{m\times n}$ captures the idea that the messages consists of small deviations about a mean, hence the model for A likes a “bouquet”. A are i.i.d. sampled from a Gaussian distribution:

(2) $$\begin{array}{c} A=[a_{1}\ldots a_{n}]\in R^{m\times n},\;a_{i}{∼}_{iid}\mathbb{N}(\mu ,\frac{\upsilon^{2}}{m}I_{m}),\\ {\left\|\mu \right\|}_{2}=1,\;{\left\|\mu \right\|}_{\infty }\leq C_{\mu }m^{-1/2}. \end{array}$$

The two assumptions on the mean force it to remain incoherent with the standard basis as m→∞.

**Assumption** **1.** *(Weak Proportional Growth). A sequence of signal-error problems exhibits weak proportional growth with parameters* $\delta >0,\;\rho \in (0,1),\;C_{0}>0,\;\eta_{0}>0$, *denoted* ${\mathrm{WPG}}_{\delta ,\rho ,C_{0},\eta_{0}}$, *if as* m→∞,

(3) $$\frac{n}{m}\to \delta ,\;\frac{{\left\|e_{0}\right\|}_{0}}{m}\to \rho ,\;{\left\|x_{0}\right\|}_{0}\leq C_{0}m^{1-\eta_{0}}\;$$

We say the cross-and-bouquet model is $\ell^{1}-\mathrm{recoverable}\;\mathrm{at}\;(I,J,\sigma )$ if for all $x_{0}\geq 0$ with supporting I and $e_{0}$ with supporting J and signs σ,

(4) $$\begin{array}{ll} (x_{0},e_{0})= & \arg\min{\left\|x\right\|}_{1}+{\left\|e\right\|}_{1}\\ & subject\;to\;Ax+e=Ax_{0}+e_{0} \end{array}$$

And the minimize is uniquely defined.

**Theorem** **1.** *For any* $\delta >0,\exists \upsilon_{0}(\delta )>0$ *such that if* $\upsilon <\upsilon_{0}$ *and* ρ<1, *in* $WPG_{\delta ,\rho ,C_{0},\eta_{0}}$ *with a distributed according to Equation (4), if the error support* J *and signs* σ *are chosen uniformly at random, then as* m→∞,

(5) $$P_{A,J,\sigma }\left[\ell^{1}-recoverable\;at\;(I,J,\sigma )\;\forall I\in \left(\begin{array}{l} \left[n\right]\\ \;k_{1} \end{array}\right)\right]\to 1$$

In other words, as long as the bouquet is sufficiently tight, asymptotically $\ell^{1}$-minimization recovers any sparse signal from almost any errors with support size less than 100%.

## 3. Improve L1 Optimization to Correct 100% of Corrupted Propagated Errors in Network Coding

### 3.1. The Variant of John and Yi’s Model

Based on Equation (2), we concatenate an identity matrix $I_{n}$ behind the matrix A. Based on the experiment, this modification does not degrade performance of the model in [21] sharply. We divide $x_{0}\in R^{n\times 1}$ into two parts: $x_{0,\;k}\in R^{k\times 1}$ and $x_{0,\;(n-k)}\in R^{(n-k)\times 1}$. If there are no errors, the coding procedure in John and Yi’s model can be expressed by

(6) $$y=\left[\begin{array}{c} A\\ I_{n} \end{array}\right]\times x_{0}=\left[\begin{array}{c} A\times x_{0}\\ I_{n}\times x_{0} \end{array}\right]=\left[\begin{array}{c} A\times x_{0}\\ x_{0} \end{array}\right]=\left[\begin{array}{c} A\times x_{0}\\ x_{0,\;k}\\ x_{0,\;(n-k)} \end{array}\right]$$

We know that the last n−k components of the $x_{0}\in R^{n\times 1}$ will remain unchanged. If $x_{0,\;(n-k)}$ is all zeros, we will know the last n−k components of y are the errors. That is to say, we can trap a part of errors. This will indirectly decrease the error rate.

### 3.2. The Organization of Data for L1 Optimization

A sketch approach is as follows: $\kappa \in R^{k\times 1}$ is the message needed to be sent where k<n. κ may not be sparse. Add n−k zeros to κ, to form $x_{0}\in R^{n\;\times \;1}$ which is sparse. Then, to get $y\in R^{m\;\times \;1}$ based on Equation (1). Divide y into two parts: A and B. The part A is sent through a secret channel. Then, the part B is sent through the networks where the network coding is performed in the relay nodes. Although adopting complex field may improve performance of network coding [29], we just consider real field rather than complex field.

L1 optimization is performed in R field. Thus, all the encodings in relay nodes are performed in R. The decoding of L1 optimization is also performed in R. All the coefficients of network coding are selected in a finite field $F_{q}$ as usual, as we do in the common network coding.

y is sent through the networks with network coding, and polluted by errors of the networks. The received messages are Y the mixture of y and errors. The max-flow min-cut C may be different from m, and usually C<<m. To adapt with transmission through networks, reorganize $y\in R^{m\times 1}$ to $y\prime \in R^{C\times \frac{m}{C}}$. This process can be expressed as follows, that is

(7) $$Y=T\times y\prime +\;T_{Z\to Y}\times Z$$

If the percentage of the “original error” is stable, the number of the “propagated errors” projected to y is the same with that of y′. For clarity and convenience, we assume that C=m in this subsection, though it is far from the truth. It is convenient for theory analysis. Therefore, we also adopt Equation (7) to analyze the case $y\in R^{m\times 1}$.

(8) $$Y=T\times y+T_{Z\to Y}\times Z$$

In reality, we have to consider the truth C<<m. In the algorithm, we assume C<<m. This simplified model captures the essence for the error spread. With some abuse of terminologies, we re-define the dimensions in Equation (8). T∈C×C, i.e., T∈m×m. T is the true transfer matrix of y. $Z\in R^{t\times 1}$ is the error vector. Note that, in our L1 optimization method, there is no need to assume t≤C/2 as done in previous works. t is arbitrarily big, and is even equal to the number of all the links and this is contradictory to intuition. However, if t is equal to the number of all the links, we can increase the sparseness of $T_{Z\to Y}$. This method will decrease the number of “propagated errors” because it is smaller than C. The propagated errors are what the t original errors are projected to the received messages Y.

Z’s all components are nonzero. t is the number of corrupted packets. $T_{Z\to Y}$ refers to the linear transform from error edges to the sink. T is C×C, $T_{Z\to Y}$ is C×t.

### 3.3. The Transfer Model in Non-Coherent Network

In the introduction we described the transfer model in the coherent network. The transfer model in the non-coherent network is different from the transfer model in the coherent network. We should clarify this model in detail because it is important for the network coding decoding. A classical random network code indicates that y includes the identity matrix as a part of each batch. The identity matrix sent by source experiences the same transform matrix T with the raw data of the batch. Thus,

(9) $$\hat{T}=\;T\cdot I+T_{Z\to Y}\cdot L$$

where $\hat{T}$ and L are the columns corresponding to I’s location in Y and Z respectively. $\hat{T}$ is C×C, and L is t×C. By substituting T into Equation (9), Equation (8) can be simplified as:

(10) $$Y=\hat{T}\cdot y+T_{Z\to Y}\cdot \left(Z-Ly\right)$$

Note that the matrix $\hat{T}$ acts as a proxy transfer matrix for T, which the sink does not know. Note that the above is mainly in reference to [25]. Equation (10) is slightly different from $Y=T\cdot y+T_{Z\to Y}\cdot Z$ which is for the coherent network. Equation (10) is for random network coding. In random network, T is unknown and it is replaced by $\hat{T}$. $Y=T\cdot y+T_{Z\to Y}\cdot Z$ is degraded from Equation (10) for random networks. In the coherent networks, there is no error in header because there are no coding vectors in the head of the packets. Therefore, L is 0 matrix.

In the sink, packets are collected until the proxy transfer matrix ($\hat{T}$) is invertible. Matrix ${\hat{T}}^{-1}$ is left multiplied in Equation (10), we get

(11) $$y={\hat{T}}^{-1}\cdot Y-{\hat{T}}^{-1}\cdot T_{Z\to Y}\cdot \left(Z-Ly\right)$$

where ${\hat{T}}^{-1}\cdot Y$ can be got, and ${\hat{T}}^{-1}\cdot T_{Z\to Y}\cdot \left(Z-Ly\right)$ is unknown. Let $y^{d}={\hat{T}}^{-1}\cdot Y$. $y^{d}$ is the result of network coding decoding in the sink. $y^{d}$ can be regarded as a deviation value of y. In principle, $\hat{T}$ can be seen as a proxy transfer matrix of the true transfer matrix T to perform decoding.

However, there is a difference of ${\hat{T}}^{-1}\cdot T_{Z\to Y}\cdot \left(Z-Ly\right)$ between $y^{d}$ and y. The difference need to be corrected through L1 optimization in [21], rather than the decoding algorithm of the traditional code. Above all, the number of “original error” is Z, and the number of “propagated error” is ${\hat{T}}^{-1}\cdot T_{Z\to Y}\cdot \left(Z-Ly\right)$. The Errors in header mentioned above is expressed by L. ${\hat{T}}^{-1}\cdot T_{Z\to Y}\cdot \left(Z-Ly\right)$, which is C×1, represents the spread result of Z. In random network coding, we just know ${\hat{T}}^{-1}$. However, $T_{Z\to Y}$, Z, L and y are all unknown. Theoretically, even if t, the number of original corrupted packets, is very small, ${\hat{T}}^{-1}\cdot T_{Z\to Y}\cdot \left(Z-Ly\right)$ also has potential to have C nonzero components. That is to say, ${\hat{T}}^{-1}\cdot T_{Z\to Y}\cdot \left(Z-Ly\right)$ pollutes every symbol of the messages $y^{d}$.

With wr(β) denote the number of nonzero components (or symbols) in an arbitrary vector or matrix β. With $wr_{norm}(\beta )\in [0,1]$ denote the normalized wr(β). If $wr_{norm}\left({\hat{T}}^{-1}\cdot T_{Z\to Y}\cdot \left(Z-Ly\right)\right)$ is 1, where the percentage of propagated errors is 100%, we cannot decode successfully with [21]. If $wr_{norm}\left({\hat{T}}^{-1}\cdot T_{Z\to Y}\cdot \left(Z-Ly\right)\right)$ is high, for example, 0.99999, John and Yi’s Model can decode successfully with a large *m* [21]. However, the information rate is very low. The above statement accords with the truth: the more errors there are, the lower the information rate is. Any method cannot contradict this basic truth. Therefore, in random networks, we can just control the sparseness of ${\hat{T}}^{-1}$ partly.

### 3.4. Formal Algorithm

**Figure 2:**
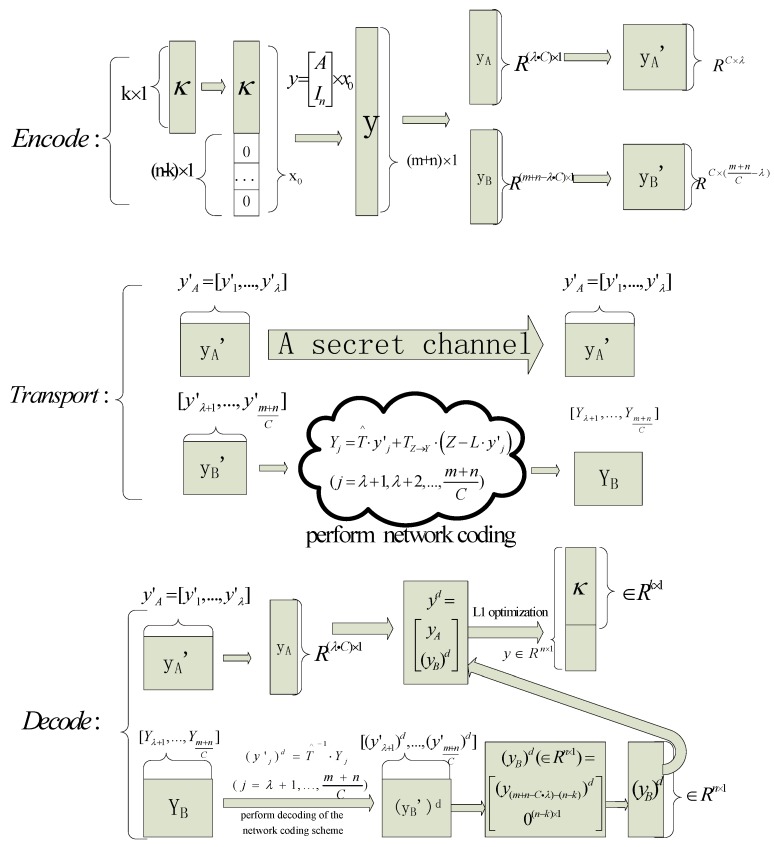
The diagrammatic sketch about Algorithm 1. The squares with white represent those added 323 zeros in the end of packets. Other graphic elements represent ordinary cases.

Here, we formally give algorithm about the correcting propagated errors in network coding via L1 optimization, and this algorithm is as Algorithm 1 which is called L1 Optimization Error Correction for Network Coding algorithm (LOECNC). κ is the message that needs to be sent. We will first give a diagrammatic sketch about the algorithm which will help us understand this algorithm more easily in Figure 2.

In Figure 2, the rectangle represents the vector while the square represents the matrix. For example, the vector $y_{B}\in R^{(m+n-\lambda \cdot C)\times 1}$ is reorganized into $y\prime_{B}\in R^{C\times (\frac{m+n}{C}-\lambda )}$. In Figure 2, $y_{B}$ is represented by a rectangle, and $y\prime_{B}$ is represented by a square. Note that C<<m, not as assumed C=m for convenience in Section 3.2. We will give an algorithm briefly to formulate this procedure.

### 3.5. The Notes on Algorithm 1

There are some notes on Algorithm 1. First, in the second step, coding equation is $y=\left[\begin{array}{c} A\\ I_{n} \end{array}\right]\times x_{0}$ other than $y=A\times x_{0}$. Second, the estimate of y is $y^{d}=\left[\begin{array}{c} y_{A}\\ {\left(y_{\left(m+n-C\cdot \lambda )-(n-k\right)}\right)}^{d}\\ {\left(y_{n-k}\right)}^{d} \end{array}\right]$. $y^{d}$ is divided into three parts: $y_{A}\in R^{(\lambda \cdot C)\times 1}$, ${\left(y_{\left(m+n-C\cdot \lambda )-(n-k\right)}\right)}^{d}$ and $0^{(n-k)\times 1}$. Among the three parts, we know $y_{A}\in R^{(\lambda \cdot C)\times 1}$ which is transmitted through a secret channel a priori, ${\left(y_{n-k}\right)}^{d}=0^{(n-k)\times 1}$ is all zeroes. Both $y_{A}$ and ${\left(y_{n-k}\right)}^{d}$ allow us to know some prior information about $y^{d}$. The ratio of $y_{A}$ and ${\left(y_{n-k}\right)}^{d}$ is (λ⋅C+(n−k))/(m+n). Even if $y^{d}$ is polluted 100% by errors, we can indirectly decrease the error rate by (λ⋅C+(n−k))/(m+n) magnitude. As long as the error rate is deceased less than 100% (is equal to 100%), we can apply the L1optimization methods in [21] to perform error correction.

Strictly speaking, the overall information rate is k/(m+n). In the model, if the error ratio is high (for example, 0.9), $x_{0}$ has to be sparse enough. In the most extreme case, there is only one non-zero component in $x_{0}$. At this point, the rate is 1/(m+n). In experiments, a good combination of parameters is m=800 and n=200. At this moment, the rate is 1/1000 which is extremely low. However, we can control the ratio of $y_{A}$ and ${\left(y_{n-k}\right)}^{d}$, and then decrease the error rate indirectly. If the fraction of errors is smaller than 0.65, the number of non-zero components in $x_{0}$ can be more. That means, the information k/(m+n) can increase fast and this model can be applied in a real environment.

What is worthy to be mentioned most is, if the original message κ itself is sparse enough, there is no need to add zeroes to it. At this moment, the normalized information rate is n/(m+n). Under the condition where m=800 and n=200, the information rate is 1/5. This is a not bad information rate in the environment where the fraction of propagated errors is 100% in random network. In the sensor network, the messages which are usually very sparse can be corrected because a characteristic data may be collected many times. Our scheme is especially suitable for above environment.

### 3.6. An Example about Algorithm 1

To aid easy understanding of Algorithm 1, we give a specific example. The most important aspect is that some parameters are set far smaller than its own real value in Algorithm 1 for the limited space. However, as an example, the essence of it is the same with Algorithm 1 though some parameters are smaller than the real value.

Step 1 Set involved parameters, among them, m=16, n=4, C=4.
Step 2 $\kappa =\left[\begin{array}{l} 1\\ 1 \end{array}\right]$, $x_{0}=\left[\begin{array}{l} 1\\ 1\\ 0\\ 0 \end{array}\right]$, $y=\left[\begin{array}{l} A\in R^{16\times 4}\\ I_{4} \end{array}\right]\times x_{0}=\left[\begin{array}{l} A\times x_{0}\\ I_{4}\times x_{0} \end{array}\right]=\left[\begin{array}{l} A\times x_{0}\\ x_{0} \end{array}\right]$. Here, $\left[\begin{array}{l} A\in R^{16\times 4}\\ I_{4} \end{array}\right]$ is the trap matrix.
  $y\;=\;{\left[1.012,\;0.986,\;1.101,\;1.014,\;1.052,\;1.022,\;1.024,0.991,\;0.957,\;0.994,\;1.003,\;0.994,\;1.017,\;0.990,\;1.006,\;1.007,\;1,\;1,\;0,\;0\right]}^{T}$.
Step 3 In a finite field $F_{q}$, perform encoding procedure of network coding scheme in every relay node. The corresponding transfer matrixes are: the message transfer matrix $\hat{T}$, the error message transfer matrix $T_{Z\to Y}$, and the error head vector transfer matrix L.
Step 4 Reorganize the vector $y\in R^{(m+n)\times 1}$ to the matrix $y\prime \in R^{C\times \frac{m+n}{C}}$, $y\prime \;=\;\left[\begin{array}{ccccc} 1.012 & 1.052 & 0.957 & 1.017 & 1.007\\ 0.986 & 1.022 & 0.994 & 0.990 & 0.997\\ 1.101 & 1.024 & 1.003 & 1.006 & 1.016\\ 1.014 & 0.991 & 0.994 & 1.007 & 1.023 \end{array}\right]$.
  Divide the matrix y′ into two parts: $y\prime =[y\prime_{A}\;;\;y\prime_{B}]$. $y\prime_{A}=\left[\begin{array}{l} 1.012\\ 0.986\\ 1.101\\ 1.014 \end{array}\right]$, $y\prime_{B}=\left[\begin{array}{cccc} 1.052 & 0.957 & 1.017 & 1.007\\ 1.022 & 0.994 & 0.990 & 0.997\\ 1.024 & 1.003 & 1.006 & 1.016\\ 0.991 & 0.994 & 1.007 & 1.023 \end{array}\right]$. Set λ = 1. Send the matrix $y\prime_{A}=\left[\begin{array}{l} 1.012\\ 0.986\\ 1.101\\ 1.014 \end{array}\right]$ through a secret channel. Send the matrix $y\prime_{B}=\left[\begin{array}{cccc} 1.052 & 0.957 & 1.017 & 1.007\\ 1.022 & 0.994 & 0.990 & 0.997\\ 1.024 & 1.003 & 1.006 & 1.016\\ 0.991 & 0.994 & 1.007 & 1.023 \end{array}\right]$ through the networks where the networks coding is performed in the relay nodes.
Step 5 $y\prime_{B}=\;[y\prime_{2},\;y\prime_{3},\;y\prime_{4},\;y\prime_{5}]\;=\left[\begin{array}{cccc} 1.052 & 0.957 & 1.017 & 1.007\\ 1.022 & 0.994 & 0.990 & 0.997\\ 1.024 & 1.003 & 1.006 & 1.016\\ 0.991 & 0.994 & 1.007 & 1.023 \end{array}\right]$, the result of network coding in relay nodes is expressed by the equation $Y_{j}=\hat{T}\cdot y\prime_{j}+T_{Z\to Y}\cdot \left(Z-L\cdot y\prime_{j}\right)\;(j=2,3,4,5)$ where $\hat{T}$ is known by the coding vector in the head of packets, but $T_{Z\to Y}$ and L are all unknown.
  $Y_{B}=\;[Y_{2},\;Y_{3},\;Y_{4},\;Y_{5}]=\left[\begin{array}{cccc} 1.123 & 0.976 & 0.817 & 1.013\\ 1.102 & 0.983 & 0.978 & 0.998\\ 1.012 & 1.012 & 1.012 & 1.023\\ 0.979 & 0.972 & 1.023 & 1.011 \end{array}\right]$ are received messages in the sink.
Step 6 Perform network coding decoding algorithm to $Y_{B}$. The result of network coding decoding is expressed by the equation ${\left(y\prime_{j}\right)}^{d}={\hat{T}}^{-1}\cdot Y_{j}\;(j=2,3,4,5)$ where ${\hat{T}}^{-1}$ is known. Because $T_{Z\to Y}\cdot \left(Z-L\cdot y\prime_{j}\right)$ is unknown, we cannot get $y\prime_{j}=(Y_{j}-T_{Z\to Y}\cdot Z)/(\hat{T}-T_{Z\to Y}\cdot L)\;(j=2,3,4,5)$. Thus, we let ${\left(y\prime_{j}\right)}^{d}={\hat{T}}^{-1}\cdot Y_{j}\;(j=2,3,4,5)$ as the estimate of $y\prime_{j}=(Y_{j}-T_{Z\to Y}\cdot Z)/(\hat{T}-T_{Z\to Y}\cdot L)\;(j=2,3,4,5)$. Then we will perform decoding with L1 optimization to get $y\prime_{j}$ based on ${\left(y\prime_{j}\right)}^{d}$. ${\left(y\prime_{B}\right)}^{d}=[{\left(y\prime_{2}\right)}^{d},\;{\left(y\prime_{3}\right)}^{d},\;{\left(y\prime_{4}\right)}^{d},\;{\left(y\prime_{5}\right)}^{d}]\;=\left[\begin{array}{cccc} 1.201 & 1.011 & 0.897 & 1.022\\ 0.902 & 0.986 & 0.978 & 1.024\\ 1.113 & 1.014 & 1.013 & 1.012\\ 0.967 & 0.972 & 0.997 & 0.977 \end{array}\right]$. ${\left(y\prime_{B}\right)}^{d}$ is the estimate of $y\prime_{B}=[y\prime_{\lambda +1},\ldots ,y\prime_{\frac{m+n}{C}}]$.
Step 7 Reorganize the matrix ${\left(y\prime_{B}\right)}^{d}\in R^{C\times (\frac{m+n}{C}-\lambda )}$ to the vector ${\left(y_{B}\right)}^{d}\in R^{(m+n-C\cdot \lambda )\times 1}$.
  ${\left(y_{B}\right)}^{d}=y\;=\;{\left[1.201,\;0.902,\;1.113,\;0.967,\;1.011,\;0.986,\;1.014,0.972,\;0.897,\;0.978,\;1.013,\;0.997,\;1.022,\;1.024,\;1.012,\;0.977\right]}^{T}(R^{16*1})$. Because $y=\left[\begin{array}{l} A\in R^{16\times 4}\\ I_{4} \end{array}\right]\times x_{0}=\left[\begin{array}{c} A\times x_{0}\\ I_{4}\times x_{0} \end{array}\right]=\left[\begin{array}{c} A\times x_{0}\\ x_{0} \end{array}\right]$, $y_{B}$ is the last 16 components of y. Respectively, the last 4 components of $y_{B}$ is $x_{0}$. However, ${\left(y_{B}\right)}^{d}$ is not equal to $y_{B}$, and it is the estimate of $y_{B}$. Thus, we cannot upload the last 4 blue components which are 1.022, 1.024, 1.012, 0.977 to 1, 1, 0, 0 where $x_{0}={\left[1,\;1,\;0,\;0\right]}^{T}$. We do not know $\kappa =\left[\begin{array}{l} 1\\ 1 \end{array}\right]$ in advance, but we know that $x_{0}=\left[\begin{array}{l} \kappa \\ 0\\ 0 \end{array}\right]$. Thus, we know 1.022, 1.024, 1.012, 0.977 will be 1.022, 1.024, 0, 0. Update
  ${\left(y_{B}\right)}^{d}=y\;=\;{\left[1.201,\;0.902,\;1.113,\;0.967,\;1.011,\;0.986,\;1.014,0.972,\;0.897,\;0.978,\;1.013,\;0.997,\;1.022,\;1.024,\;0,\;0\right]}^{T}(R^{16*1})$
  The numbers with blue are components which correspond to the known numbers in 408 which can be considered as the prior knowledge. The numbers with pinkish red are 409 components which correspond to the unknown numbers in $x_{0}$.
Step 8 In the sink, receive matrix $y\prime_{A}=\left[\begin{array}{l} 1.012\\ 0.986\\ 1.101\\ 1.014 \end{array}\right]$ through the secret channel. Denote the vector $y_{A}=\left[\begin{array}{l} 1.012\\ 0.986\\ 1.101\\ 1.014 \end{array}\right]$.
Step 9 Reorganize the vector $y_{A}\in R^{(\lambda \cdot C)\times 1}=R^{4\times 1}$ and the vector ${\left(y_{B}\right)}^{d}\in R^{(m+n-C\cdot \lambda )\times 1}=R^{16\times 1}$ into $y^{d}\in R^{20\times 1}=\left[\begin{array}{c} y_{A}\\ {\left(y_{B}\right)}^{d} \end{array}\right]$. The first 4 red components are sent by the secret channel, and the last 2 components of $y^{d}$ are trapped by the trap matrix $\left[\begin{array}{l} A\in R^{16\times 4}\\ I_{4} \end{array}\right]$ which is constructed specially. $y^{d}=\;{\left[1.012,\;0.986,\;1.101,\;1.014,1.201,\;0.902,\;1.113,\;0.967,\;1.011,\;0.986,\;1.014,0.972,\;0.897,\;0.978,\;1.013,\;0.997,\;1.022,\;1.024,\;0,\;0\right]}^{T}$.
  $y\;=\;{\left[1.012,\;0.986,\;1.101,\;1.014,\;1.052,\;1.022,\;1.024,0.991,\;0.957,\;0.994,\;1.003,\;0.994,\;1.017,\;0.990,\;1.006,\;1.007,\;1,\;1,\;0,\;0\right]}^{T}$. Thus, the green components are polluted. Up on $y^{d}$, in the real field R, perform L1 optimization of John and Yi’s scheme to get y. Because we already know 1.012, 0.986, 1.101, 1.014 and 0, 0 of y, we can certainly decoding y with John and Yi’s scheme which can recover 100% of the corrupted observations where the corrupted ratio is (20 − 6)/20 = 70%. $x_{0}=\left[\begin{array}{l} \kappa \\ 0\\ 0 \end{array}\right]=\left[\begin{array}{l} 1\\ 1\\ 0\\ 0 \end{array}\right]$ is decoded successfully. The numbers with green are components which correspond to the known numbers in $y^{d}$ which can be considered as the prior knowledge. The numbers with pinkish red are components which correspond to the unknown numbers in $y^{d}$.
Step 10 Select the first k symbols of y as κ, that is $\kappa =\left[\begin{array}{l} 1\\ 1 \end{array}\right]$. We recover the original message successfully.

### 3.7. Compressed Header Overhead

**Figure 3:**
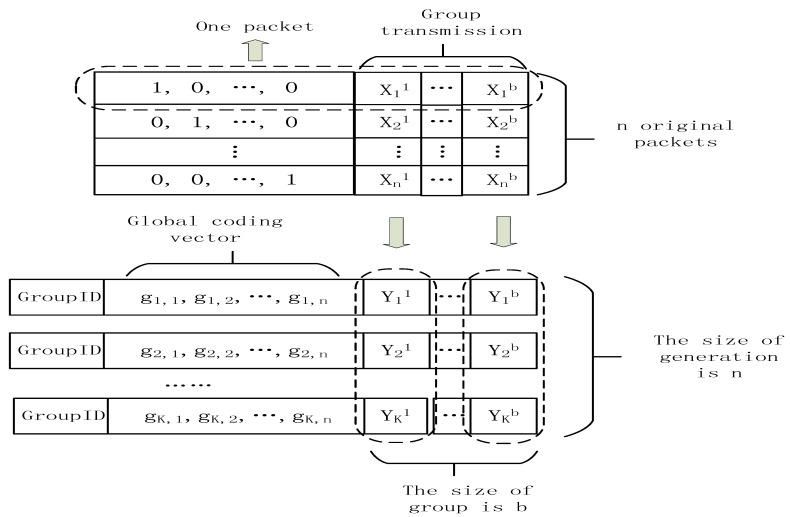
The packet format of practical network coding.

The header overhead in network coding is a very important issue in Algorithm 1 because this algorithm mainly copes with the environment of random network coding. The header overhead problem is very relevant to our scheme. There are two main methods to decrease the header overhead: the block transmission and the compressed header overhead. Chou gives the format about random network coding. The procedure of network coding in Figure 3 can be expressed as the following equation.

(12) $$\left[\begin{array}{ccc} Y_{1}^{1} & \ldots & Y_{1}^{b}\\ Y_{2}^{1} & \ldots & Y_{2}^{b}\\ \ldots & \ldots & \ldots \\ Y_{K}^{1} & \ldots & Y_{K}^{b} \end{array}\right]=T\cdot \left[\begin{array}{ccc} X_{1}^{1} & \ldots & X_{1}^{b}\\ X_{2}^{1} & \ldots & X_{2}^{b}\\ \ldots & \ldots & \ldots \\ X_{n}^{1} & \ldots & X_{n}^{b} \end{array}\right]=\left[\begin{array}{cccc} g_{1,1} & g_{1,2} & \ldots & g_{1,n}\\ g_{2,1} & g_{2,2} & \ldots & g_{2,n}\\ \ldots & \ldots & \ldots & \ldots \\ g_{K,1} & g_{K,2} & \ldots & g_{K,n} \end{array}\right]\cdot \left[\begin{array}{ccc} X_{1}^{1} & \ldots & X_{1}^{b}\\ X_{2}^{1} & \ldots & X_{2}^{b}\\ \ldots & \ldots & \ldots \\ X_{n}^{1} & \ldots & X_{n}^{b} \end{array}\right]$$

In this equation, the transfer matrix from the source to the sink node is $\left[\begin{array}{cccc} g_{1,1} & g_{1,2} & \ldots & g_{1,n}\\ g_{2,1} & g_{2,2} & \ldots & g_{2,n}\\ \ldots & \ldots & \ldots & \ldots \\ g_{K,1} & g_{K,2} & \ldots & g_{K,n} \end{array}\right]$ which is denoted as T. The decoding can be performed successfully if and only if K≥n and T is invertible. In Figure 3, the size of generation is n, and the size of block is b. The definition of generation and block are referred in [30,31]. The cost of network coding scheme is the overhead of transmitting extra symbols in each packet. If we increase the size of block, which is the number of symbols about messages in a packet, the normalized overhead can be reduced. However, in Algorithm 1, we cannot increase the size of block without limit because the $b=\frac{m+n}{C}=\frac{m+n}{n}$. If we increase the size of b, we have to increase $\frac{m}{n}$. As illustrated in above paragraph, we know that the optimal value of $\frac{m}{n}$ is 4. If $\frac{m}{n}$ is not equal to 4, the effectiveness of L1 optimization will reduce. Thus, though the method of block transmission has certain effectiveness, we cannot take advantage of this method unlimitedly.

Another method is to compress header overhead, about which there are many works [32,33]. Among them, the latest important work about compress header overhead is [33] which is very interesting and useful. Gligoroski and so on use compressed sparse row (CSR) technique to reduce the header overhead [33].

## 4. Find the Optimal Number and Optimal Positions of Relay Nodes in Network Coding with Social Networks

In Algorithm 1, we always assume the matrix Y in $Y=\hat{T}\cdot y+T_{Z\to Y}\cdot \left(Z-Ly\right)$ is full rank. However, in the actual environment, the matrix Y may not always be full rank which is the famous “all-or-nothing” problem in random network coding [26]. If *Y* is not full rank, the effectiveness of Algorithm 1 will be undermined greatly. Certainly, we can keep receiving fresh packets from the network until *Y* is full rank. Thus, if we consume more time and energy to receive more packets until Y is full rank, the procedure of Algorithm 1 can be done unaffectedly. However, it will consume too many resources such as the energy and the time to receive more packets until Y is full rank. Therefore, we must additionally find a method to hedge the consumed resource resulting from receiving more packets until Y is full rank. For a certain network, when all the relay nodes perform network coding, assume the number of packets which are received ceaselessly until Y is full rank which is ϒ. A possible method is to choose only a part of relay nodes other than all of them to perform network coding while not increasing the value of ϒ. Fewer relay nodes, which perform the network, will certainly result in less energy consumption. It is a difficult to find the optimal number and optimal positions of relay nodes. Because the topology is unknown and the coefficients of network coding change over time, it is obvious that we would be better to adopt decentralized algorithm to find the optimal relay nodes. The social characteristic of the relay nodes in network coding inspires us to adopt the theorem of social networks. There are some “key” relay nodes which can transmit more “informative” messages to downstream nodes. The reputation-based trust model of social network research field will help us to find the upstream relay nodes that have more opportunities to have informative for any time other than one time. Next, we will discuss this novel scheme. This scheme is a supplement to Algorithm 1. It will improve the performance of Algorithm 1 though Algorithm 1 can work without this supplementary scheme.

### 4.1. “All-or-Nothing” Problem about Network Coding in WSN

Full rank of received packets is required to invert the linear mapping so as to recover the transmitted data packets. This requirement unfortunately results in a key drawback of network coding: either all of the packets in a session are recovered simultaneously or none can be recovered, which leads to long delays and low throughputs. Long delays and low throughputs are especially unbearable in WSN which has limited battery energy. If the packets received in the sink are not full rank, the successful decoding probability of L1 optimization will decline though it also has a great chance for decoding successfully.

### 4.2. Overcome “All-or-Nothing” Problem with Reputation-Based Trust Model of Social Network

A wireless sensor network is treated as a social network where the sensors are the main entities which are referred to as human beings in a traditional social network. In wireless sensor networks, the nodes resemble individuals in the way that they communicate with their peers. Nodes of the sensor network have their own social life, and based on that assumption, we leverage ideas from social networks to show how the nodes can communicate in a “social networking” style to achieve significant efficiency. When some common rules of social networks are applied in WSN, the performance of WSN will receive a significant reduction of overhead traffic leading to longer battery life of embedded nodes and better utilization of the network [22].

**Figure 4:**
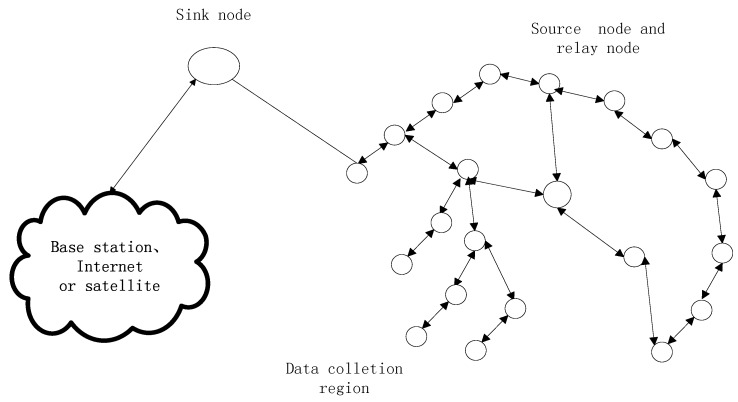
No cluster head architecture of WSN.

In this paper, to simplify our model, we adopt no cluster head architecture of WSN which is illustrated in Figure 4. Although WSN with cluster head architecture is more universalistic, it is complicated to perform network coding in WSN with cluster head architecture [34]. The roles of the common relay node and the cluster head are different in sense of network coding. The common relay node and the cluster head are in the different hierarchy in the architecture. They cannot perform network coding in the same hierarchy. If we perform network coding in WSN with cluster head architecture, a complicated network coding scheme with two hierarchies has to be proposed. To simplify our model, we just adopt no cluster head architecture of WSN which will help us understand our scheme more easily. In Figure 4, the common sensor nodes in the data collection region include source nodes and relay nodes. In Figure 4, we highlight which node is the source in multicast network, and any common node has the potential to be the source node. The messages received by sink nodes are sent to the base station, Internet or the satellite.

#### 4.2.1. Stastical Trust Based on the Rank of Packets in the Downstream Nodes in WSN

First, we should give a clear definition of “trust” in our model. There are many trust models in social networks and wireless sensor networks [35]. The metrics to measure the social characteristic of a social node are, for example, the consumed energy, connection frequency and successful transmission. In our model, the trust is defined as the number of the times that packets in the downstream nodes are full rank. The more the trust value of a relay node, the more the chance that the relay node has the potential to be a “key” relay node which will transmit more “informative” messages to downstream nodes.

#### 4.2.2. Collecting Experiences to Build the Trust for an Intermediate Node in WSN

Every relay node will send ACKs of the full rank report to the h hops upstream nodes. Every relay node will receive some ACKs of the full rank report from h hops downstream. How does one judge the packets of a relay node that has full rank of received packets? Because the topology is variable, we cannot fix the incoming edges for a relay node, therefore, we cannot judge what time the rank of received packets for a relay node is full. In this model, we define “the full rank” as the “max rank” of the received packets. Then, we select combinations of packets which has the minimum of packets and is full rank. For example, the max flow min cut is 10, then the dimension of NEC is also 10. For a relay node, the rank of all the received packets is 5. We think the “full rank” is 5 (not 10) for this relay node. We select the combinations whose number is minimum among all the combination of the received packets. We can certainly find a combination of 5 incoming edges whose received packets are full rank. Then, the nodes will send ACKs along the corresponding incoming edges to all the h hops upstream nodes.

After a period of time Γ, every relay node will have a record about the times of its own received ACKs. The times of its own received ACKs for a relay node will be treated as its own trust value. The value of Γ and h can be set according to the situation. The bigger the value of h, the more precise the trust value. If h is set as the number of hops from source to the sink, there is no need to use the trust model of social network which is based on probabilistic method rather than deterministic method. In this case, the network coding scheme is optimal and there is no longer the so-called “all-or-nothing” problem. However, the big h will consumed too much resource which is unacceptable for WSN. Generally, for a common WSN, h is set as 2 which is based on the next experiment results. It is enough to make reputation-based trust of the relay node while the h is set as 2.

The reputation-based trust of a relay node reflects the reality about socialistic characteristic in network coding. The factors reflecting the trust value for a relay node come from two aspects: the topology and the random coefficients of network coding scheme. The two factors produce the randomness. If the two-factor producing randomness is fixed in a manner, the reputation-based trust value will really reflect the degree of importance for a relay node which makes the received packets of the downstream nodes full rank. Although the topology for some kinds of WSN, for example, underwater wireless sensor networks, is variable, it tends to stay stable for a period of time. Another randomness coming from the random network coding can be restrained. Every node just randomly generates a local coding kernel for itself, and will perform network coding with the first time local coding kernel for the next time. Thus, the topology and local coding kernel are all stable. The reputation-based trust value will reflect the social characteristic for a node, and we can use the concepts of social networks to research the “all-or-nothing” problem of network coding in WSN.

**Figure 5:**
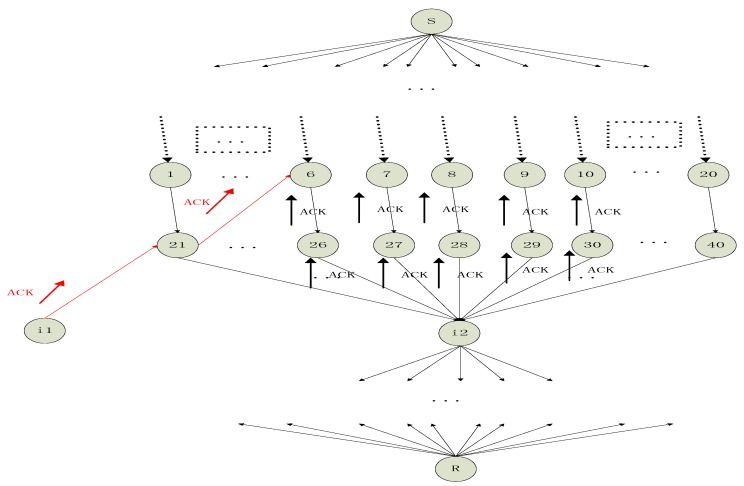
The building procedure of reputation-based trust about an intermediate node. The node i1 is another node which is similar to i2. The red ACKs are part of all the ACKs which are sent by the i1.

Figure 5 is an example of the above scheme. For the relay node 6, we want to find its reputation-based trust value. During time Γ, the relay node 6 will keep receiving ACKs from its downstream relay nodes. For simplicity, we only give its two downstream nodes which are nodes i1 and i2. The details about node i1 are omitted and we focus on node i2. The max flow min cut of this network is 10. For node i2, it has many incoming edges and we assume there are 20 incoming edges. After computing, we know the rank of all the received packets is, for example, 5. Then, we select a combination of 5 incoming edges whose received packets are full rank. The 5 packets span a vector space whose rank is 5. i2 will send 5 ACKs along the 5 incoming edges upstream to h hops away, and in this case h is set as 2. The upstream 5 relay nodes which is h hops away are nodes 6, 7, 8, 9, 10. With respect to node i2, nodes 6, 7, 8, 9, 10 have increased its own reputation-based trust value while nodes 1, 2, 3, 4, 5 and 11–20 have not increased its own reputation-based trust value. Only considering the case this time, we say nodes 6, 7, 8, 9, 10 are more likely to send “informative” packets downstream than the nodes 1, 2, 3, 4, 5 and 11–20. Similarly, node 6 also receives ACKs from its downstream node i1 which is 2 hops away, and node 6 also increase its own reputation-based trust value one time. After time Γ, every relay node has a reputation-based trust value. The bigger the reputation-based trust value of a node, the more chance that this node is a “key” relay node which will transmit more “informative” packets to its downstream nodes. The node i1 is another node which is similar to i2. The red ACKs are part of all the ACKs which are sent by the i1.

#### 4.2.3. Network Coding Based on Reputation-Based Trust

In the stage of network coding which is based on reputation-based trust, we will set a threshold value for the trust value. The relay nodes with trust value larger than the threshold value will perform network coding. On the contrary, the relay nodes with trust value smaller than the threshold value will not perform network coding and go into hibernation. The above scheme will select some nodes as active nodes and other nodes as hibernation nodes which will save energy.

**Figure 6:**
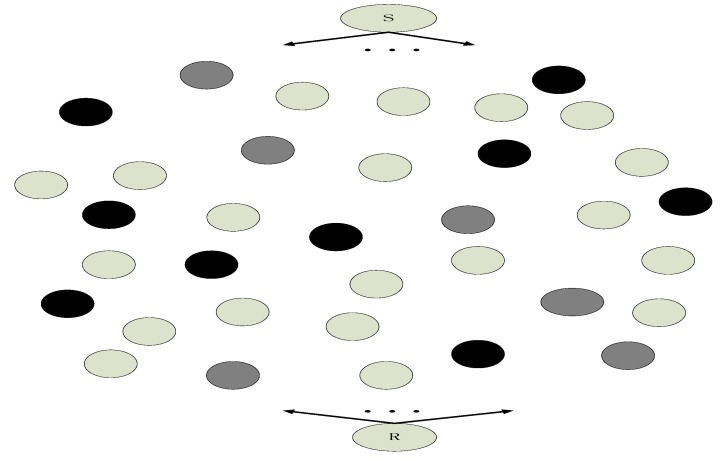
Parts of intermediate nodes performing network coding in WSN. The highest trust value nodes with black, the median value nodes with gray, and the lowest trust value nodes with the white.

In Figure 6, the trust values are divided into three levels: the highest trust value in the relay nodes with the most black color, the median value in the relay nodes with the light black color, and the lowest trust value in the relay nodes with the white color. Only the relay nodes with the most black color will perform network coding. Compared with the situation where all the relay nodes perform network coding that situation where only a part of nodes perform network coding will save energy. In the sense of network coding, the relay nodes with high trust value are similar with the active nodes in social network.

Theoretically, this model does not always select the optimal nodes, which in reality will be the “key” nodes. Because the scheme which is based on social network theory is completely decentralized and distributed, we cannot always reach the ideal situation: the selected nodes with highest trust value will help completely overcome the “all-or-nothing” problem in network coding. However, many works about social networks illustrate that, if the trust model is defined reasonably, the model which is based on social network theory really can reach an acceptable result even if the model is decentralized and distributed. In our work, the definition about “trust”, which is also illustrated in Figure 5, is really a reasonable and novel model which captures the point of the “all-or-nothing” problem in random network coding. The following experiment results confirm our conclusion.

## 5. Experimental Section

We will first give the experiment results about L1 optimization combined with the secret channel in Section 3. Because L1 optimization mainly refers to the scientific computation, we use MATLAB as the experiment tool. Then, we will give the experiment results about the error correction in WSN which is based on L1 optimization and social networks method with OMNET++.

### 5.1. Experiments about L1 Optimization with MATLAB

#### 5.1.1. The Propagation Behavior of Original Errors

**Figure 7:**
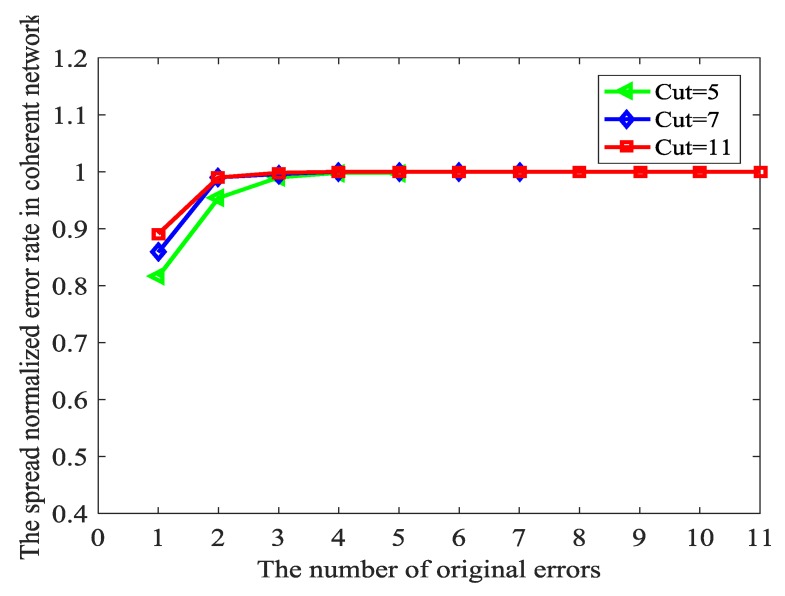
The spread of errors in the coherent networks.

As mentioned in the above subsection, in this section we will see how the network coding affects the propagation of the original errors. Although this work mainly refers to non-coherent network, how the error spreads in the coherent network is also a beneficial referential experience to the study of non-coherent network. For coherent networks, Z is affected by $T^{-1}\cdot T_{Z\to Y}$, which is shown in Figure 7. It demonstrates that the propagated errors cannot pollute all the received messages because both are in a small network coding field which results from small max-flow-min-cut and few errors. The greater the original errors, the greater the propagated errors, which is compatible with the truth. Because the network is a priori, for few original errors, there is potential to stop the spread of errors by constructing $T^{-1}\cdot T_{Z\to Y}$ meticulously. In the coherent network, we can construct $T^{-1}\cdot T_{Z\to Y}$ because the topology is known by us. Thus, if the number of original errors is smaller than C, we can make the number of the propagated error $T^{-1}\cdot T_{Z\to Y}\cdot Z$ is smaller than C.

For non-coherent networks, in ${\hat{T}}^{-1}\cdot T_{Z\to Y}\cdot \left(Z-Ly\right)$, we only can select ${\hat{T}}^{-1}$ other than $T_{Z\to Y}\cdot \left(Z-Ly\right)$ in the sink. In the sink, we construct the matrix T after receiving C coding vector. Thus, T is an exogenous variable. Naturally, ${\hat{T}}^{-1}$ is also an exogenous variable. Although we cannot construct ${\hat{T}}^{-1}$, we can select such packets whose coding vectors in the head make ${\hat{T}}^{-1}$ is sparse. This will indirectly decrease the number of ${\hat{T}}^{-1}\cdot T_{Z\to Y}\cdot \left(Z-Ly\right)$. However, it is time-consuming to select such packets whose coding vectors in the head make ${\hat{T}}^{-1}$ sparse. If this method of selecting packets is not adopted and the size of coding field is small, the number of nonzero components in ${\hat{T}}^{-1}\cdot T_{Z\to Y}\cdot \left(Z-Ly\right)$ is perhaps smaller than C. With the size of coding field becoming bigger, the number of nonzero components in ${\hat{T}}^{-1}\cdot T_{Z\to Y}\cdot \left(Z-Ly\right)$ will be equal to C. That is to say, the propagate errors pollute all the received messages in the sink. We will investigate how the original error spreads in the non-coherent network through the experiment. Assume $wr\left(T_{Z\to Y}\cdot \left(Z-Ly\right)\right)=C$, which is also the worst situation. The above statement means, if there is no interface to ${\hat{T}}^{-1}$, the errors will be propagated to the whole network.

**Figure 8:**
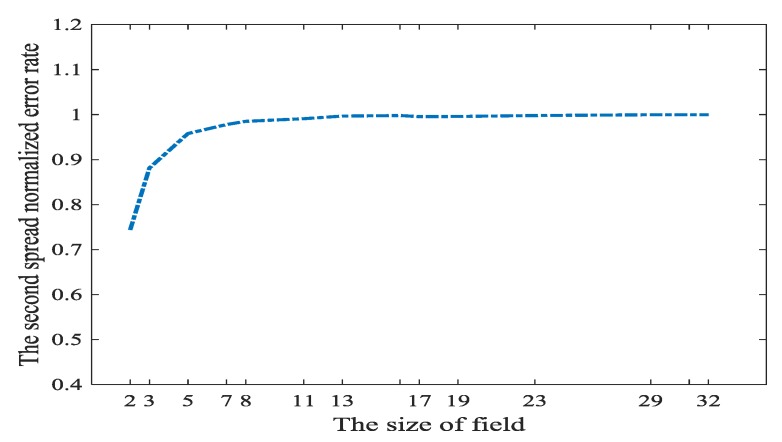
The spread of errors in non-coherent networks on field size. The blue line is the function of spread normalized error rate based on the size of field.

Figure 8 shows, if we randomly select coefficients of the local coding kernel, the received messages are nearly all polluted. However, when the size of network coding field is smaller than 7, some received messages are not polluted. Theoretically, the bigger the size of coding finite field, the more the opportunities that a symbol in this field is nonzero. Therefore, ${\hat{T}}^{-1}$ will be very dense if the size of coding field is big.

In the network coding field whose size is smaller than 7, we can randomly construct network coding coefficients to apply the L1 optimization in [21]. However, if the coding field is smaller than 7, it cannot to provide $\hat{T}$ is full rank with high probabilities. If a big network coding field is adopted, ${\hat{T}}^{-1}$, i.e., has to be constructed delicately.

Through the above experiments we can see the situation about the error spread in the network coding is serious. Especially, in random network coding, the propagated error ${\hat{T}}^{-1}\cdot T_{Z\to Y}\cdot \left(Z-Ly\right)$ always pollutes all the received messages when coding field is bigger than 7. Thus, we have to face such pessimistic fact and propose an effective method to confront such a situation.

#### 5.1.2. The Effect of L1 Optimization in Network Random Coding

We will investigate the performance of the Algorithm 1. The ratio of the messages sent through the secret channel is denoted as ϑ. $\left\|x_{0}\right\|$ is on behalf of the sparseness of vector $x_{0}$. In Algorithm 1, if the components of the original uncompressible messages κ have no zeroes, $\left\|x_{0}\right\|=k/n$. In Figure 6, we set $\left\|x_{0}\right\|=1$.

Most of the parameters are the same as the simulation in [21]. The parameters are as follows. υ=0.05, δ=0.25. m∈{100, 200, 400, 800}. We have not got any channel to get the original implement details of [21]. Because some implement details may be different, our effectiveness is a little worse. However, the whole trend is the same.

**Figure 9:**
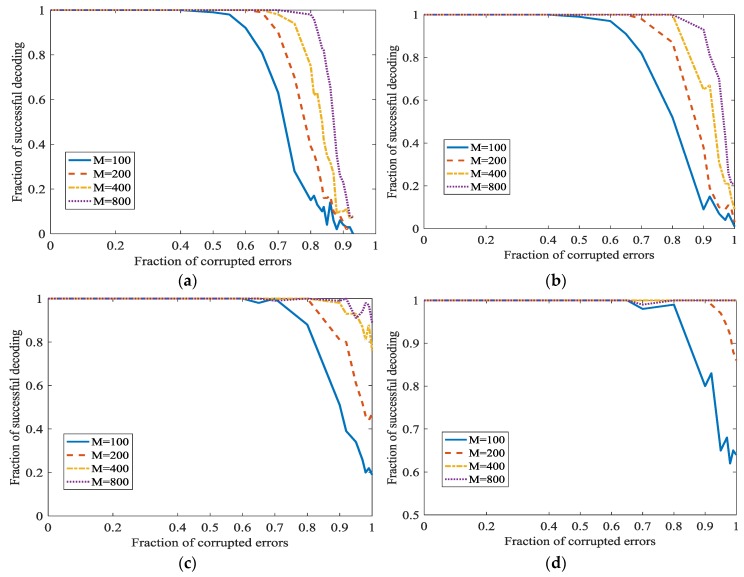
Error correction in L1 optimization when $\left\|x_{0}\right\|=1$ at different ϑ. (**a**) ϑ=0; (**b**) ϑ=0.1; (**c**) ϑ=0.2; (**d**) ϑ=0.3.

In Figure 9, $\left\|x_{0}\right\|=1$ and ϑ is set as 0, 0.1, 0.2 and 0.3 respectively in different subfigures. In Figure 9a, we can see that the percentage of successful recovery and the fraction of corrupted errors are in inverse proportion. When m increases, the correcting fraction τ also increase, and almost approaches 1. This point is surprising and attractive. It can be natural to adopt this L1 optimization for correcting the dense propagated errors in network coding. In Figure 9a, even 0.95 density errors can be recovered. However, the successful correction fraction is not satisfactory when errors density is high, for example, 0.95. However, we can increase m to increase the successful correction ratio. Generally, when m = 800 and fraction of errors is 0.6, the fraction of successful correction approaches 1. This certainly can meet the need in real communication. However, $\left\|x_{0}\right\|=1$ also means a low information rate here. However, when the fraction of corrupted errors is 100%, this algorithm cannot recover the original uncompressible messages. In Figure 9b–d, the secret channel is used. With the increase of ϑ, we can correct more dense errors. Especially, when the fraction of propagated errors is 100%, we can also correct it.

**Figure 10:**
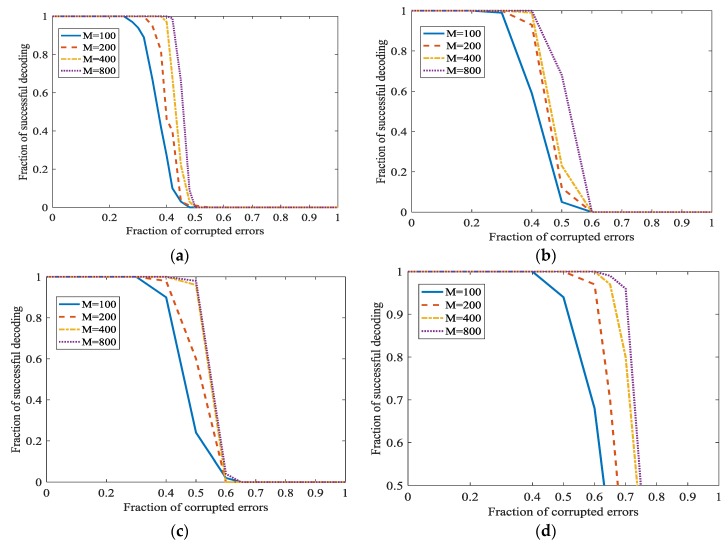
Error correction in L1 optimization when $\left\|x_{0}\right\|=m^{1/2}$ at different ϑ. (**a**) ϑ=0; (**b**) ϑ=0.1; (**c**) ϑ=0.2; (**d**) ϑ=0.3.

If $\left\|x_{0}\right\|=1$, the information rate will be very low. We will also investigate the performance of the Algorithm 1 at different $\left\|x_{0}\right\|$. In Figure 10, $\left\|x_{0}\right\|=m^{1/2}$. The high fraction of successful decoding is at the cost of the low information rate. If we want to increase the information rate, the fraction of successful decoding will be down. However, even when the information rate is higher, L1 optimization also has a surprisingly high fraction of corrected errors. In traditional codes, the fraction of corrected errors is 0.5 at most when the information rate approaches 0. The fraction of successful decoding is approximately 0.47. It is also higher than traditional codes, i.e., to a considerable information rate.

Both $\left\|x_{0}\right\|=1$ and $\left\|x_{0}\right\|=m^{1/2}$ are extreme situations. It would be better to keep a balance between error-correcting ratio and information rate. It shows, when $\left\|x_{0}\right\|$ increases, i.e., higher information rate, the fraction of correction becomes lower. However, the fraction of correction is also acceptable.

#### 5.1.3. Set Other Parameters to Increase the Information Rate

**Figure 11:**
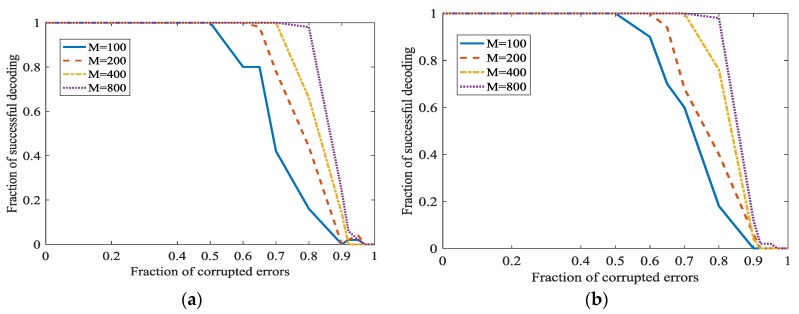

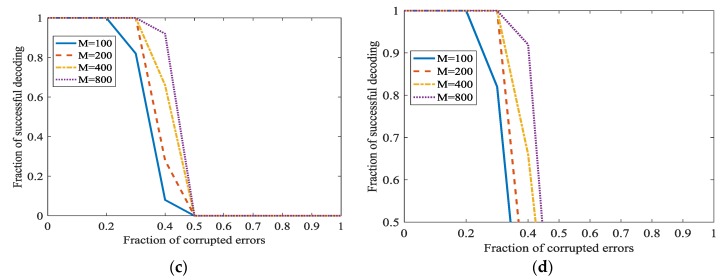
Error correction in L1 optimization at different m/n and $\left\|x_{0}\right\|$. (**a**) $\left\|x_{0}\right\|=1$ and m/n=3; (**b**) $\left\|x_{0}\right\|=1$ and m/n=2; (**c**) $\left\|x_{0}\right\|=m^{1/2}$ and m/n=3; (**d**) $\left\|x_{0}\right\|=m^{1/2}$ and m/n=2.

In [21], a better parameter about m is as m∈{100, 200, 400, 800}. A bigger m/n will increase the fraction of successful decoding. However, when m/n is bigger, the information rate will become smaller. We will investigate the performance of the Algorithm 1 when m/n is smaller. In Figure 11, m∈{100, 200, 400, 800} and n≈m/3 or n=m/2. Figure 8 shows that a bigger m/n will indeed increase the fraction of successful decoding. However, the information rate has a considerable increase while the decrease of the fraction of successful decoding is acceptable. In Figure 8, the secret channel is not used (ϑ=0) in order to investigate L1 optimization more clearly.

**Figure 12:**
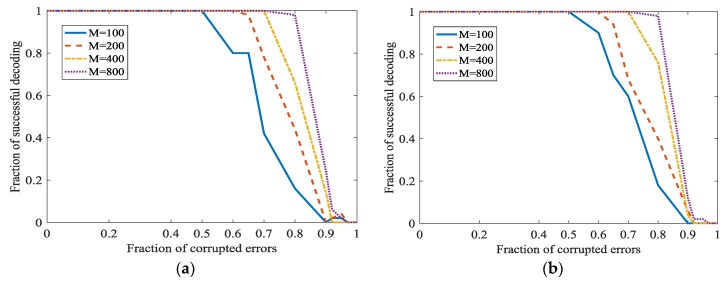
Error correction in L1 optimization at different ϑ when m/n decrease. (**a**) ϑ=0.1; (**b**) ϑ=0.3.

In Figure 12, the secret channel is used. $\left\|x_{0}\right\|=1$, m∈{100, 200, 400, 800} and n≈m/3. Figure 12 shows that even m/n is not the optimal value 4, adopting the secret channel in L1 optimization also achieves a good performance.

Table 1 lists the important terms used in the experiment, which is useful for understanding the parameters used in the experiment.

#### 5.1.4. The Time for L1 Optimization

**Figure 13:**
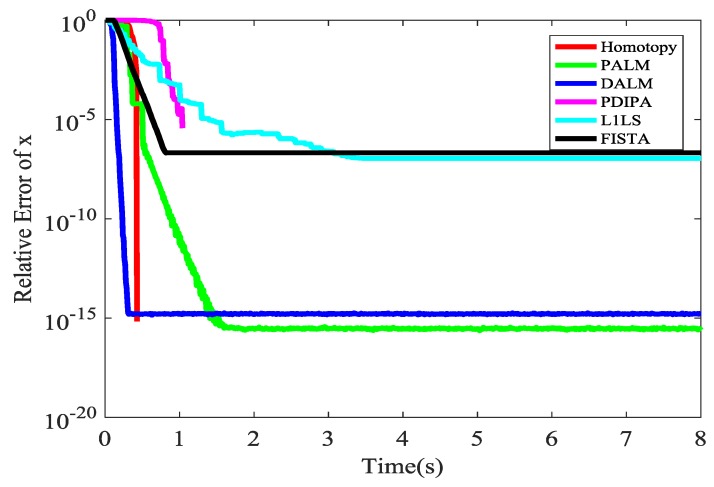
The time of different kernel L1 optimization.

Our algorithm includes a sub-algorithm, which is a concise optimization algorithm. There are many available L1 optimization sub-algorithms. These kernel algorithms will be compared on aspect of the time consuming. We will choose the fastest L1 optimization sub-algorithm as the kernel of our algorithm. They are compared as shown in Figure 13. This mainly refers to [36]. The concise meaning of the abbreviations in the legend are also shown in this webpage [36]. The abbreviations in the legend refer to different L1 optimization. It is easy to see that Homotopy algorithm is the fastest algorithm and it is adopted in our algorithm.

Generally speaking, when L1 optimization is applied to communications, the primary concern is that the computation consumes too much. However, we will show, compared with the decoding algorithm of traditional codes, the L1 optimization is also very efficient in time. Our scheme is compared with a (256,128) Low Density Parity Check Code (LDPC) in time consuming. LDPC is fast and generally adopted in a real industrial environment. It is a traditional block code and has efficient decoding algorithm in time. (256,128) means the normalized information rate is 1/2, the minimum distance is 64. Its most tolerant error density is 0.25, so we assume the error fraction is less than 0.25. Given a network with max-flow-min-cut 6 (<7), we perform network coding in the finite field with size 7. A 128-symbols long message is coded with LDPC, and then gets through the network performing network coding in relay nodes. Finally, we decode the 128 messages in the sink. This simulation is performed on a common PC, rather than GPU or FPGA. From Table 2, we can see our algorithm is also efficient in terms of the time consumed.

### 5.2. Experiments about the Error Correction in WSN Based on L1 Optimization and Social Networks Method

**Figure 14:**
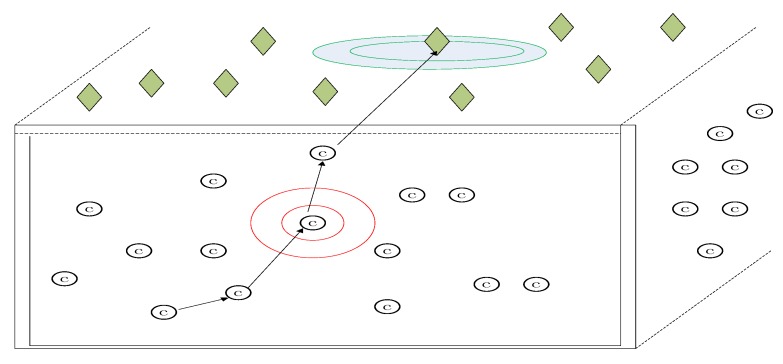
The architecture of underwater acoustic sensor networks. The sink node in the green circle and the sensor node in the red circle are the examples with special emphasis.

We will give the experiment results about the error correction in a WSN environment based on L1optimization and social networks method with OMNET++. The WSN is an underwater acoustic sensor network whose network structure is shown in Figure 14. The green rhombus is the sink node. The red circle is the common sensor node. The sink node in the green circle and the sensor node in the red circle are the examples with special emphasis. Underwater acoustic sensor networks are a typical application of WSN. Underwater acoustic sensor networks have the following properties: (i) limited bandwidth capacity and high propagation delays which is due to the low speed of sound; (ii) the underwater acoustic channel is severely impaired; (iii) high bit error rates and temporary losses of connectivity; (iv) underwater sensors are prone to failures; (v) batteries are energy constrained and cannot be recharged [37]. Thus, the underwater acoustic sensor network is in severe need of transmission methodology like our scheme which can increase the information rate and correct dense error. The underwater acoustic sensor network is a typical scenario of WSN which will be a good test for our scheme.

The experiment environment is as follows. There are 100 sensor nodes and 1 base station. Sensor nodes are randomly distributed on the approximately 1000 square meter roof. The communication distance for a sensor node is 100 m. The finite field is $GF(2^{8})$ which is a common field for network coding. The nodes will send ACKs along the corresponding incoming edges to all the h hops upstream nodes. h is set as 2. We employed two metrics to evaluate the performance of wireless sensor networks. SDR is the number of successful delivered packets over the total packets sent by the source node, the normalized energy consumption is equal to total energy consumption divided by SDR. Three schemes are compared from the perspective of SDR and normalized consumed energy. The three schemes are traditional network coding, network coding which is based on only L1 optimization, and network coding which is based on L1 optimization and social characteristic. In L1 optimization, the fraction of original messages by secret channel is set as ϑ=0.1.

**Figure 15:**
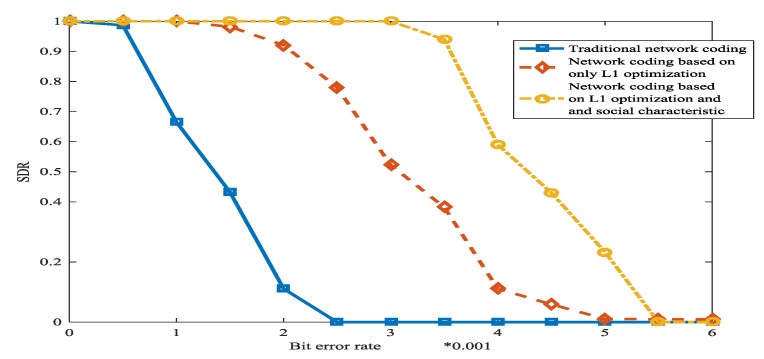
The successful delivered packets based on the bit error rate in WSN.

Figure 15 shows network coding which is based on L1 optimization and social characteristic has the most powerful error correction ability. The reason is that L1 optimization and the method which is based on social network are very effective.

**Figure 16:**
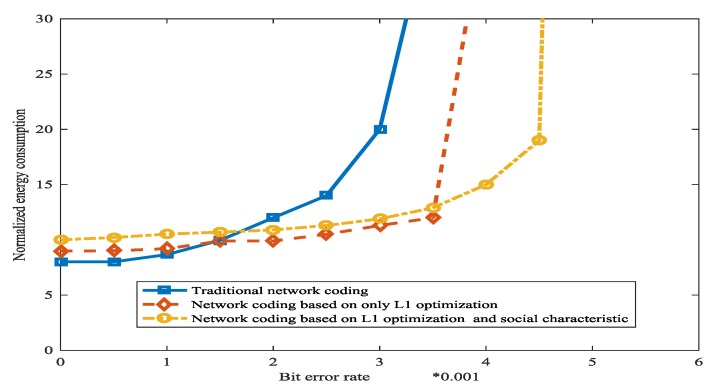
The normalized consumed energy based on the bit error rate in WSN.

Figure 16 shows the comparison between the consumed energies with three schemes. When the bit error rate is low, traditional network coding has the lowest consumed energy while network coding which is based on L1 optimization and social characteristic has the most consumed energy, because the operation for the L1 optimization and social network method will consume energy. However, as the bit error rate increases, the benefits of L1 optimization and social network method are being realized, and network coding which is based on L1 optimization and social characteristic has the least consumed energy.

## 6. Conclusions

We propose a new framework of the network error correction for random network coding in WSN. The scheme combines two methods which are L1 optimization and social networks to correct the propagated dense errors for the random network coding in WSN. Using the secret channel and the trap matrix methods, our scheme successfully overcomes the shortage of original L1 optimization which cannot be propagated errors polluting exactly 100% of received packets. Based on the method of social networks, we also propose a new distributed approach that establishes reputation-based trust to overcome the “all-or-nothing” problem. The latter social network method further increases the successful decoding probability of the former L1 optimization method. The two methods of L1 optimization and social networks coordinate with each other and successfully overcome the shortcoming that the traditional block codes can correct corrupted errors no more than *C*/2 in random network coding. Experiment results show that even if the error rate in WSN is very high, our scheme can also perform network coding to increase the network throughput of WSN. Our scheme is also efficient in time. As far as we know, our scheme is the only scheme which can correct the dense propagated errors for network coding. Our scheme has great significance to wireless sensor networks which usually have high error rates, limited battery energy and can be badly in need of network coding to increase the information rate and prolong the lifetime of WSN.

## Figures and Tables

**Table 1:** Terms used in the experiment.

Variable	Definition
*m*	The number of rows of coding matrix $A\in R^{m\times n}$
*n*	The number of columns of coding matrix $A\in R^{m\times n}$
$\left\|x_{0}\right\|$	0 norm
*ϑ*	The fraction of messages sent by the secret channel

**Table 2:** Comparison of times.

Algorithm	Time (s)
LDPC	0.7527
Our	0.6285
